# Associations between parents’ subjective time pressure and mental health problems among children in the Nordic countries: a population based study

**DOI:** 10.1186/s12889-015-1634-4

**Published:** 2015-04-10

**Authors:** Hrafnhildur Gunnarsdottir, Ylva Bjereld, Gunnel Hensing, Max Petzold, Lene Povlsen

**Affiliations:** Department of Public Health and Community Medicine, Section of Social Medicine, Sahlgrenska Academy at University of Gothenburg, Gothenburg, Sweden; Department of Nursing, Health and Culture, University West, 461 86 Trollhättan, Sweden; Department of Social Work, University of Gothenburg, Gothenburg, Sweden; Centre for applied biostatistics, Department of Public Health and Community Medicine, Section of Occupational and Environmental Medicine, Sahlgrenska Academy at University of Gothenburg, Gothenburg, Sweden; Unit for Health Promotion Research, University of Southern Denmark, Esbjerg, Denmark

**Keywords:** Time pressure, Parents, Child mental health, Strength and difficulties questionnaire, Nordic countries

## Abstract

**Background:**

The home, the family and the parents represent a context of everyday life that is important for child health and development, with parent-child relationships highlighted as crucial for children’s mental health. Time pressure is an emerging feature of modern societies and previous studies indicates that parents with children living at home experience time pressure to a greater extent than people with no children living at home. Previous studies of children’s mental health in relation to parents’ time pressure are lacking. Hence, the purpose of this study was to examine the association between parents’ subjective time pressure and mental health problems among children in the Nordic countries as well as potential disparities between boys and girls in different age groups.

**Methods:**

4592 children, aged 4-16 from Denmark, Finland, Norway and Sweden, participating in the 2011 version of the NordChild study, were included. The Strength and Difficulties Questionnaire was used to measure children’s mental health and associations to parents’ time pressure were assessed by multiple logistic regression analysis.

**Results:**

Among children of parents experiencing time pressure, 18.6% had mental health problems compared to 10.1% among children of parents experiencing time pressure not or sometimes. The odds of mental health problems were higher among both boys (OR 1.80 95% CI 1.32-2.46) and girls (OR 1.95 95% CI 1.42-2.66) if their parents experienced time pressure when adjusted for financial stress. The highest prevalence of mental health problems in the case of parental time pressure was found among girls 13-16 years old (23.6%) and the lowest prevalence was found among boys 13-16 years old (10.7%).

**Conclusions:**

In this study an association between parents’ subjective time pressure and increased mental health problems among children was found. Given that time pressure is a growing feature of modern societies, the results might contribute to an explanation as to mental health problems are common among children in the Nordic countries in spite of otherwise favourable conditions. Additional research on the linkage between parents’ experienced time pressure and children’s and adolescents’ mental health problems is needed to confirm the novel findings of this study.

## Background

The context of the present study is the welfare state of the Nordic countries where family policies supporting dual-earner/dual-carer families are prominent. The essence of the dual-earner/dual-carer model consists of favourable parental leaves and generous day care services, which enable parents to share paid work and care of children [1,2]. Implicit in the dual-earner/dual-carer model is a need to balance roles and responsibilities in the work and family domains, which can be complicated and cause conflicts [3]. Women’s participation in the labour market is high in the Nordic countries and women still assume the main responsibilities for housework even though the disparities between men and women’s total amount of time spent in work (paid and housework) have declined throughout the last decades. Men are taking a larger share of housework responsibilities than ever before, while mothers in all of the Nordic countries are still devoting more time to childcare than fathers [4-7]. Time pressure evolves if there is a discrepancy between the standards and/or the ambitions of what people want to or have to do within the domains of everyday life and the realisations of these. If time pressure is perceived as out of control or chronic it gets problematic and threatening to health [8]. Time pressure is a complex feature of modern societies [8] that has emerged as a considerable social problem [9] and has become a common theme in popular discourse [10]. It concerns the organisation of social practices within a certain time unit [8,10] and its emergence has been related to economic, cultural and technological changes of society [9,10]. Related to economic changes, time efficiency in production has become crucial for economical profit; leading to consistent competition to achieve the largest possible output per time unit [9]. This often results in employees working long hours, working faster and being accessible and flexible [10]. Technological changes characterising modern societies have made multi-tasking a natural pattern that has led to intensified social practices [8]. Cultural changes towards the idea of “the good life” consisting of doing as much as possible during a lifetime have resulted in individuals attempting to do everything as fast as possible [9].

Two dimensions of time pressure have been highlighted as important to recognize, one objective and one subjective [11]. The objective dimension embraces the crude lack of time, a quantifiable event and possibly controllable, that can be captured by time diaries where people measure their time allocation in different fields of everyday life. The subjective dimension embraces the experience of time pressure as feelings of fragmented time, demands to do things faster and constantly being rushed [11]. Previous research indicates that parents with children living at home experience time pressure to a greater extent than others [12,13]. Previous studies have as well indicated that parents of young children perceive time pressure to be challenging to health lifestyles of their children and family [14,15] and that time pressure can have negative effects on parental mental health [16,17]. Parents’ experiences of time pressure have previously been found to be associated with lack of support, financial stress, long working hours and parenting younger children [18].

The home, family and parents, represent a context of everyday life that is crucial for child health and development [19]. Bronfenbrenner [20,21] conceptualises this in his ecological model of human development, which embraces the structural systems affecting the individual and the family. The family as a unit and its intra-familial processes represent the microsystem in Bronfenbrenner’s model, which is to a great extent influenced by processes in the mesosystem, the other settings in which the family members are participating (e.g. schools for children), as well as the exosystem level, which are the settings in which members are not directly participating (e.g. parents work for children) [21]. The macrosystem level then represents the cultural and political environment embracing the other systems and between all the systems a reciprocal interaction exists [20]. Drawing upon Bronfenbrenner’s model, the emerging time pressure in modern societies can be considered not only of importance for parental mental health but children’s as well.

**Figure 1:**
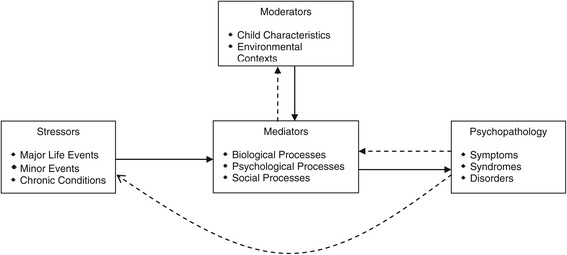
General conceptual model of the role of stressors in the aetiology of child and adolescent psychopathology from Grant et al. [24].

Children’s mental health problems embrace a broad range of behavioural, emotional and mental disorders and are currently considered a main public health concern. Estimating the global prevalence of mental health problems is challenging due to differences in methods and measurement, however, referencing studies of representative samples of children with psychiatric diagnoses, Kieling et al. [22] concluded that mental health problems affect 10-20% of children and adolescents worldwide. Moreover, mental health problems among children and adolescents in the Nordic countries are considered common [23], in spite of the family friendly policies. The aetiology of children and adolescents’ mental health problems is complex, embraces a range of individual, familial and societal factors [22] and needs to be considered from various perspectives, one of which is the parents’. Grant et al. [24] propose a general conceptual model of the role of stressors in the aetiology of child psychopathology (Figure 1). The model illustrates how stressors (embracing a broad range of stressful experiences, from daily hassles to traumatic life events) contribute to child and adolescent psychopathology through moderating factors and mediating processes [24].

To our knowledge no previous studies have investigated the relationship between parents’ time pressure and children’s mental health problems. However, previous studies have found associations between other parent-related stressors, such as high parental stress [25] and work-family conflicts [26] and mental health problems among children. Previous studies have also indicated that girls react more negatively to stressors than boys [27-29] and that the gender differences in stress reactions generally appear during adolescence [30,31].

Drawing on the view of time pressure as an emerging social problem contributing to tensions in children’s ecological systems of development and a gap in the research area, the aim of our study was to examine the association between parents’ perceived time pressure and children’s mental health problems. The focus was on the subjective dimension of time pressure and attempts were made to capture an aggregated picture of the conditions of families with children in the Nordic welfare states by using data from four Nordic countries (Denmark, Finland, Norway and Sweden). The broad age span (4-16 years) of participants also enabled analysis of gender differences in different age groups.

## Methods

### Participants

The present study was part of the 2011 version of the Nordic Study of Children’s Health and Wellbeing (NordChild) which is a cross-sectional survey conducted in the five Nordic countries Denmark (DK), Finland (FI), Iceland (IS), Norway (NO) and Sweden (SE). Ethical approval was achieved according to the prescribed guidelines in each of the Nordic countries. In DK the National Committee on Health Research Ethics, in FI the National Committee on Medical Research Ethics (TUKIJA), in IS the National Bioethics Committee, in NO Regional Committee for Medical and Health Research Ethics, REC South Easth and in SE the Regional Board of Ethical Vetting in Gothenburg.

Stratified sampling based on age and gender was made from the total population of children aged 2 – 17 years old and consisted of approximately 3000 children from each country, randomly sampled from the strata. A postal questionnaire addressed to the child’s primary care giver was used and the overall response rate after reminders was 48.8%.

The present study’s population consisted of parents of children 4-16 years old from DK, FI, NO and SE (n = 4952) who answered a question about their experience of time pressure in everyday life. Of the parents who answered the questionnaire (considered themselves the child’s primary caregiver) 83.0% were mothers, 14.6% fathers and 2.4% others/not.

### Measurements

#### Mental health problems

The American Academy of Paediatrics (AAP) [32] defines mental health problems as behavioural or emotional signs or symptoms that cause impairment but do not necessarily meet the diagnostic criteria for a mental health disease/psychiatric diagnosis. They further describe that the term encompasses neurodevelopmental, psychological, socio-emotional problems as well as substance abuse and adjustment to stressors. Moreover, mental health problems can also embrace psychosomatic symptoms as fatigue, headaches, eating disorders and functional gastrointestinal symptoms [33]. The aspects of children’s mental health problems measured in this study were hyperactivity, emotional, peer and behavioural problems captured by the parental version of Strengths and Difficulties - Total Difficulties Score (SDQ-TDS), which previously has been found a comprehensive measurement of overall mental health problems among children 4-16 years old [34-37]. The Strengths and Difficulties Questionnaire (SDQ) is a widely used instrument [38,39], which has been translated and found valid for use in all the Nordic countries [40] and was included in the 2011 version of the NordChild survey. The SDQ consists of 25 items covering emotional, peer and behavioural problems as well as hyperactivity and pro-social behaviour divided into five subscales [38]. Each subscale generates a score ranging from zero to 10. The SDQ-TDS is the sum of the hyperactivity, emotional problems, conduct problems and peer problems scales, generating a scale score ranging from zero to 40 [38]. The SDQ-TDS has been found suitable for screening for mental health problems among children in normal populations and found to correspond sensitively with clinical diagnoses of psychiatric disorders [41]. As recommended [38,42] the 90^th^ percentiles were used as cut-offs for mental health problems. Previous studies have observed gender and age specific differences in the scoring on the SDQ [35,42]. Hence the cut-offs were calculated for each gender and the age groups: preschool children (4-6 years old), primary school children (7-12 years old) and adolescents (13-16 years old). Children scoring over the cut-off point of their gender and age group were categorised as having mental health problems.

#### Parents’ subjective time pressure

The subjective dimension of time pressure was assessed by the question, “Do you feel rushed when keeping up with the duties of everyday life?” previously used in studies of time use and time pressure [13,43]. Response alternatives were: Yes most often, Yes sometimes or No. Drawing upon Garhammer’s [8] definition of time pressure becoming problematic when experienced out of control or chronic, the exposure of interest was parents reporting feelings of rush ‘most often’. Thus the answers were dichotomised to ‘most often’ and ‘no/sometimes’ in the analysis and parents answering ‘most often’ are hereafter referred to as experiencing time pressure or time-pressured.

#### Potential confounding variables

The choice of covariates of interest was made by theoretical selection among variables possibly influencing both parents’ subjective time pressure and child mental health status. Bullying victimisation was considered important because of it being a strong predictor of the child’s mental health [44,45] and possibly increasing parents’ experience of time pressure, measured by the question, “Is your child being bullied?” (Yes often/sometimes or No/don’t know). Child family and living conditions are widely recognised determinants of child health [46] and previous studies have found them to be associated with parents’ experienced time pressure [9,18]. Family and living conditions were assessed by family financial stress (inability to pay ordinary bills and/or lack of cash reserves), parents’ educational level (university level or lower educational level), parents’ civil status (married/cohabiting or single parent), and parents’ working hours per week (<37 hours, 37-40 hours or > 40 hours). Parents’ health/wellbeing was considered a possible confounding factor and was assessed by reported sick leave (long term = more than 60 days during the last twelve months). Furthermore, child long-term illness (LTI) was considered a possible confounder and defined as one or more modest or severe physical symptoms presented for at least three months throughout the last year.

### Statistical analysis

SPSS version 20.0 was used for the statistical analysis. The mean TDS and the proportion of children with mental health problems (scoring above the 90^th^ percentile) were calculated among children of parents experiencing time pressure as well as children of parents who were not/sometimes experiencing time pressure.

A bivariate logistic regression analysis was performed to measure the association between parents’ subjective time pressure and children’s mental health problems in total as well as age and gender specific. Then a stepwise logistic regression model was built to adjust for covariates considered potential confounders. In the first step each covariate’s effects on the parameter estimate were assessed. If resulting in changes in parameter estimates for parents’ time pressure > 10% the covariate was included in the final model. The final regression model was used to calculate the adjusted odds ratios (OR) for the whole sample as well as for age and gender specifically. Additionally the final regression model was used to calculate the adjusted ORs for the whole sample of children in each country.

## Results

Country specific characteristics of the participating children are shown in Table 1.

**Table 1:** **Country specific characteristics of participants**

	**Denmark**	**Finland**	**Norway**	**Sweden**	**Nordic countries**
	**(n = 1314)**	**(n = 1181)**	**(n =1288)**	**(n =1169)**	**(n = 4952)**
	**n (%)**	**n (%)**	**n (%)**	**n (%)**	**n (%)**
Gender:					
Boy	669 (50.9)	579 (49.0)	654 (50.8)	593 (50.7)	2495 (50.4)
Girl	645 (49.1)	602 (51.0)	634 (49.2)	576 (49.3)	2457 (49.6)
Age:					
4-6 years	335 (25.5)	297 (25.1)	290 (22.5)	276 (23.6)	1198 (24.2)
7-12 years	607 (46.2)	585 (49.5)	612 (47.5)	544 (46.5)	2348 (47.4)
13-16 years	372 (28.3)	299 (25.3)	386 (30.0)	349 (29.9)	1406 (28.4)
Mean SDQ-TDS (SD)^a^	7.7 (4.3)	7.9 (4.1)	7.3 (4.2)	7.3 (4.2)	7.6 (4.2)
Parents experiencing time pressure:					
Most often	50 (3.8)	224 (19.0)	169 (13.1)	255 (21.8)	698 (14.1)
Not/sometimes	1264 (96.2)	957 (81.0)	1119 (86.9)	914 (78.2)	4254 (85.9)
Parents reporting financial stress					
Yes	244 (18.6)	399 (33.8)	243 (18.9)	232 (19.8)	1118 (22.6)
No	1053 (80.1)	763 (64.6)	1019 (79.1)	919 (78.6)	3754 (75.8)

^a^SDQ-TDS = Strengths and Difficulties - Total Difficulties Score, SD = Standard Deviation.

A statistically significant difference in the prevalence of mental health problems was observed among children of parents who experienced time pressure (18.6%) compared with children of parents who did not or sometimes experience time pressure (10.1%), see Table 2. When stratified by age and gender the highest prevalence of mental health problems in the case of parental time pressure was found among girls 13-16 years old (23.6%) and the lowest prevalence was found among boys 13-16 years old (10.7%). The adolescent boys of time-pressured parents did not have higher prevalence of mental health problems when compared to adolescent boys of parents without time pressure.

**Table 2:** **Mean and standard deviations of total difficulties score, cut-off points and prevalence of mental health problems**

	**Age group**	**Cut-off**	**Time pressure**			**No time pressure**		
			**n**	**TDS**	**MHP** ^**b**^ **%**	**n**	**TDS**	**MHP %**
				**Mean (SD)** ^**a**^			**Mean (SD)**	
All children		-	698	8.7 (4.8)	18.6^a^	4254	7.4 (4.1)	10.1^c^
Boys	Total	-	364	9.0 (5.0)	17.5	2131	7.7 (4.2)	9.5
	4-6 years old	14	104	9.5 (4.2)	16.5	514	8.0 (3.9)	8.8
	7-12 years old	14	187	9.3 (5.3)	21.1	993	7.8 (4.3)	10.7
	13-16 years old	13	73	7.5 (4.9)	10.7	624	7.2 (4.1)	9.6
Girls	Total	-	334	8.4 (4.6)	17.8	2123	7.1 (3.9)	9.2
	4-6 years old	12	92	8.6 (3.9)	22.0	488	7.2 (3.5)	12.0
	7-12 years old	13	170	8.1 (4.8)	16.6	998	7.1 (4.1)	9.6
	13-16 years old	13	72	8.6 (5.2)	23.6	637	6.9 (4.0)	7.6

^a^TDS = Total Difficulties Score, SD = Standard Deviation.

^b^MHP = Mental Health Problems.

^c^difference between time pressure and no time pressure significant using Chi2 test *p* ≤ .001.

The regression analysis showed that both boys and girls had higher odds of mental health problems if parents were experiencing time pressure compared with parents who did not or sometimes experience time pressure. When adjusted for financial stress (the only confounding variable affecting the parameter estimate >10%), boys had 1.80 (95% CI 1.32-2.46) times higher and girls 1.95 (95% CI 1.42-2.66) times higher odds of mental health problems if their parents experienced time pressure (Table 3). The odds of mental health problems were higher among children of both time pressured mothers (OR 2.07, 95% CI 1.64-2.62) and fathers (OR 1.46, 95% CI 0.75-2.84). When analysed stratified by country, increased odds of mental health problems were found among children to time pressured parents in all the Nordic countries; Denmark OR 2.31 (95% CI 1.18-4.54), Finland OR 2.05 (95% CI 1.38-3.05), Norway OR 2.00 (95% CI 1.26-3.20) and Sweden OR 1.82 (95% CI 1.21-2.73). When analysed by age groups, the results indicated the strongest association between parents’ time pressure and mental health problems among boys 7-12 years old and girls 13-16 years old (Table 3) but when tested as the interaction time pressure*age*gender the differences in ORs could not be confirmed statistically significant.

**Table 3:** **Associations between children’s mental health problems and parents’ subjective time pressure by gender and age groups**

**Age group**		**Mental health problems**			
		**n**	**OR (95% CI)** ^**a**^	**n**	**Adj OR** ^**b**^ **(95% CI)**
			**reference: no time pressure**		**reference: no time pressure**
All children		4923	2.03^***^ (1.63-2.52)	4850	1.87^***^(1.50-2.33)^c^
Boys	Total	2479	1.94^***^ (1.43-2.63)	2442	1.80^***^ (1.32-2.46)
	4-6 years	614	2.05^*^ (1.12-3.74)	606	1.68 (0.89-3.16)
	7-12 years	1174	2.23^***^ (1.48-3.34)	1157	2.25^***^ (1.48-3.41)
	13-16 years	691	1.12 (0.52-2.45)	679	0.99 (0.45-2.20)
Girls	Total	2444	2.13^***^ (1.57-2.89)	2408	1.95^***^ (1.42-2.66)
	4-6 years	575	2.07^*^ (1.17-3.65)	566	2.05^*^ (1.15-3.63)
	7-12 years	1163	1.88^**^ (1.19-2.97)	1148	1.55 (0.96-2.50)
	13-16 years	706	2.75^***^ (1.51-5.03)	694	2.67^**^ (1.45-4.93)

^a^OR = Odds Ratio, CI = Confidence Interval.

^b^adjusted for financial stress, other covariates tested for as a confounder: Bullying victimisation, long term illness, parents’ marital status, educational level, working hours, long term sick leave.

^c^Hosmer and Lemeshow test *p* = .904, Cox & Snell R^2^ .028, Nagelkerke R^2^ .055.

^*^ *p* < .05 ^**^ *p* < .01 ^***^ *p* ≤ .001.

## Discussion

The main finding of the present study was that children of parents who experienced time pressure ‘most often’ had higher odds of mental health problems than children of parents who did not or sometimes experience time pressure. To our knowledge no previous studies have investigated this association but the findings are consistent with studies of other parent-related stressors, indicating that high parental stress [25,47] as well as work-family conflict [26] are associated with mental health problems among children.

Since, previous studies of the association between parents’ time pressure and children’s mental health problems is lacking, we used Grant’s et al. [24] general conceptual model of the role of stressors in the aetiology of child and adolescent psychopathology and Bronfenbrenner’s [20,21] ecological model of human development to explain our results and the hypothesised direction of the association observed.

**Figure 2:**
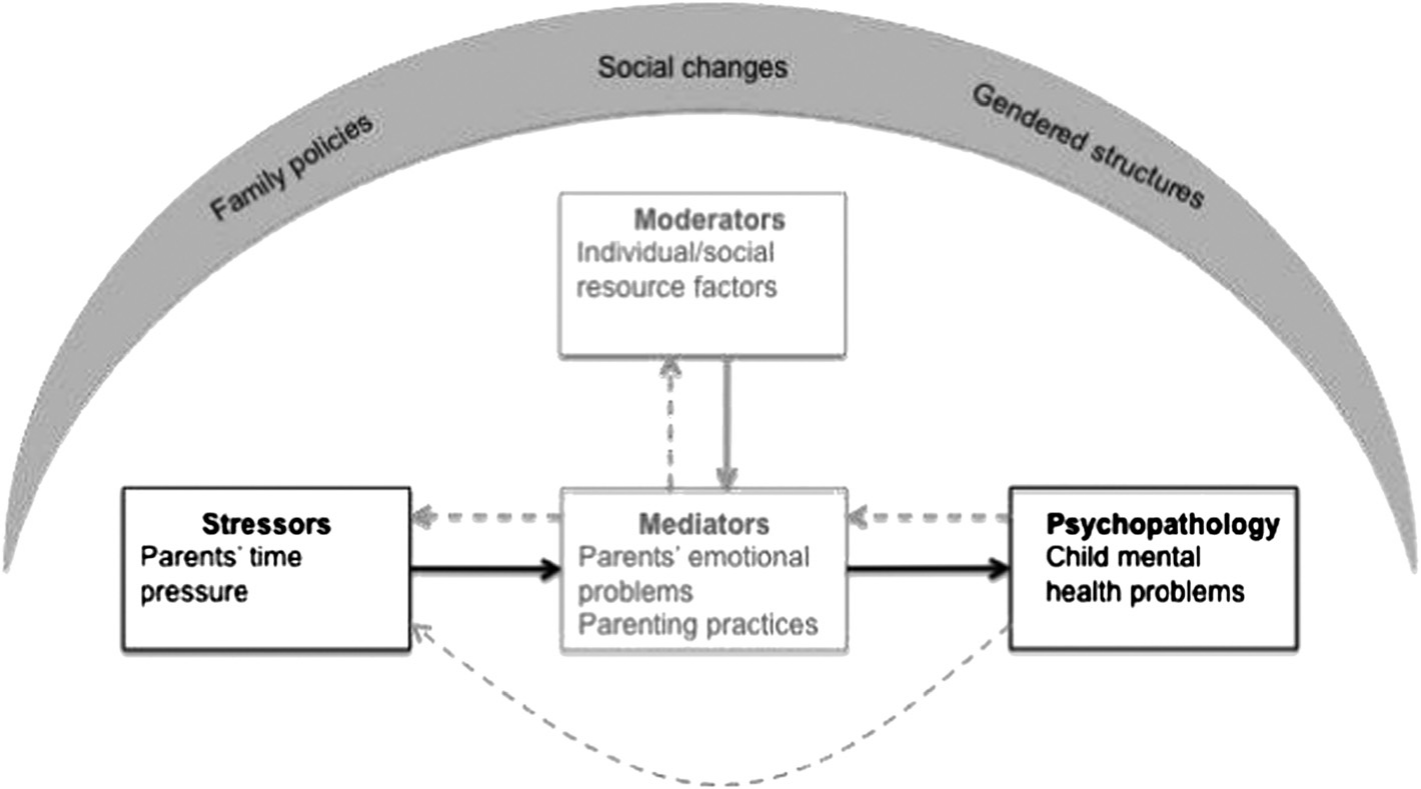
The theoretical underpinnings of the study and the hypothesised directions of the relationships between the variables illustrated in Grant’s et al. conceptual model [24]. (Variables and hypothesised directions of associations studied are illustrated in black text and arrows while other variables considered important as well as other potential directions of relationships not studied are illustrated in gray text and arrows).

Parents’ experience of time pressure is not necessarily completely related to crude lack of time, rather, we propose it to be considered a symptom of tensions in parents’ everyday life. Whether these tensions originate from work life, family life, or parents’ own or societal expectations, they are examples of processes in children’s external ecological systems that according to Bronfenbrenner’s ecological model [20,21] affect health and development. As such parents’ perceived time pressure, can be considered a potential stressor in children’s and adolescents’ everyday life, which according to our results is associated with mental health problems. The mechanisms between parents’ perceived time pressure and children’s mental health problems are most certainly complex. In Figure 2 the theoretical underpinning of the study and the hypothesised direction of the observed association are illustrated in relation to Grant’s et al. conceptual model. Moderating and mediating factors potentially important for the mechanisms between parents’ time pressure and children’s mental health problems are illustrated in a gray scale as they were not possible to analyse in the present study but are considered important to study in future research. The structural aspects (context and conditions) considered important in relation to the observed associations are also illustrated as overarching in relation to the occurring mechanisms, similarly to the macrosystem of Bronfenbrenner’s ecological model.

Time pressured parents might not be able to communicate the warmth, consideration and accessibility considered crucial for children’s and adolescents’ mental health [48,49]. Even more so since time pressure can lead to stress/emotional problems among parents [16,17] and typical symptoms of stress are withdrawal and attempts to reduce cognitive and emotional demands. As parental emotional well-being and parenting practices have previously been proposed as potential mechanisms through which low economic status is associated with children’s mental health problems [50], it is reasonable to assume that these are also important mediators in the associations between parents’ time pressure and child mental health problems.

In Grant’s et al. model, child characteristics and environmental context are proposed as moderators that influence the relation between stressors and psychopathology. Previously studies have highlighted individual and social resource factors (e.g. child’s personality, good family atmosphere, supportive family network, social support and recreational activities) as important in counteracting mental health problems in case of stress and adversity [47,51]. Future research of the association between parents’ time pressure and children’s mental health problems should include such factors.

The emergence of time pressure as a social problem has been related to economic, cultural and technological changes in society [9,10]. These changes are highly apparent in the everyday life of families in the Nordic countries where the dual-earner/dual-carer model has become the norm [1,52]. Many Nordic families need two breadwinners to manage regular expenses and the generous day-care system makes it possible even for single parents to combine work and childcare. The context of the present study was the welfare states of the Nordic countries but it can be assumed that parents’ experiences of time pressure are even more of a challenge in countries with less supportive family policies.

The high degree of gender equality is a successful product of the Nordic welfare system, at least in theory, but maybe not as successful in practice. Women’s/mothers’ labour market participation has become a norm while fathers’ participation in child care is still struggling to become an accepted norm, as illustrated by fathers’ perceptions of breaking the societal norms when choosing to work part-time to take care of their children [53]. Such a discrepancy can be a source of imbalance manifested as time pressure. Another possible source of such imbalance is the labour market’s demands of effectiveness, accessibility and flexibility, challenging the work-life parity. Further, expectations and ideals emerging in parallel with social changes of modern societies [8] add to an imbalance in everyday life and the experience of time pressure. In congruency with previous research [15,26], the results of the present study therefore emphasise the importance of considering child mental health in a wide perspective of the structural and social relations shaping the context of children’s everyday life.

### Gender perspectives

In our estimations of the prevalence of mental health problems among children exposed to parental time pressure we identified gender differences among adolescents (13-16 years old). While the prevalence was almost the same among those exposed to parental time pressure and those not exposed among adolescent boys, the prevalence of mental health problems among adolescent girls was almost three times higher if their parents experienced time pressure. As mentioned before, previous studies of associations between parental time pressure and children’s mental health are lacking, not to mention studies about gender differences in such associations. Theories on gender as a social structure and ongoing activity embedded in social interactions [54,55] might shed light on the observed difference in the prevalence of mental health problems between adolescent boys and girls. According to such theories, the social norm of femininity entails caring and taking responsibility for relationships, implying that the parents might raise more demands and implicit expectations on girls to take responsibility for themselves, assist with the household and/or take care of eventual younger siblings, in order to ease the parents’ hectic everyday life. Boys, on the other hand, may not be equally expected to carry out tasks related to care and the household and thus not as burdened by their parents time pressure. On the other hand, there might also be potential differences in how adolescent boys and girls express symptoms of mental health problems. As the constructed norms of masculinity often expect boys to be stoic and strong [55], adolescent boys might not express the negative emotions they experience related to the time-pressured parent. In a previous study children aged 10, 13 and 15 years old demonstrated an awareness of social expectations of boys to react to both physical and psychological problems with stoicism and strength [56]. Likewise, parents might not be as prone to recognising the symptoms expressed by boys, as it doesn’t fit the perceived norm of masculinity. Hence, if interactions are gendered in such a way between the time-pressured parent and the adolescent boy there still might be a possibility that the boys were experiencing problems related to their parents’ time pressure even though not observed in the estimated prevalence. Even though gender differences in the associations between parents’ time pressure and children’s mental health problems could not be confirmed statistically significant, the results of this study support that children’s mental health problems needs to be considered in the perspective of prevailing gender structures, both when addressed in research and practice.

### Methodological considerations

The main strength of the present study is the possibility to identify intergenerational health linkages as the data consists of information gathered simultaneously about Nordic parents and children in a broad age span. This has the potential to contribute to an increased understanding of the intertwined lives of parents and children. At the same time it should be highlighted that all data was reported by parents, which means that the assessment of children’s symptoms was based on the parents’ awareness and reporting of the symptoms. However, the parental version of the SDQ has been widely used and found reliable in screening for child mental health problems [57].

The main limitation of the study is the low response rate. A comparison of the study sample to the general population confirmed an underrepresentation of single parents and parents with low educational levels (Table 4). Moreover, it is likely that the prevalence of parents’ experiencing time pressure are underestimated in the study as time pressure can be considered a logical reason for not completing an extensive postal questionnaire. The main focus of the study was to study association rather than prevalence. This may reduce limitations due to the low response rate, as studies of association have been found less sensitive to non-response biases than prevalence estimations [58,59]. Important to bear in mind when interpreting and discussing results is to make a distinction between time pressure and stress. The question used was not measuring stress among parents; it was measuring parents’ experience of time pressure, which can be seen as an exposing factor likely to lead to stress. Due to the cross-sectional design of the study, no conclusions about causality can be made, just as the possibility of reciprocal associations between parents’ time pressure and children’s mental health are important to bear in mind. Family relationships are reciprocal in nature and thus it cannot be excluded that parenting a child with mental health problems may contribute to an experience of time pressure, which also Grant et al. argue is relevant for relations among stressors, moderators, mediators and psychopathology in general.

**Table 4:** **Characteristics of the answering parents (NordChild2011 2-17years old) compared to general population**

	**Denmark**				**Finland**				**Iceland**				**Norway**				**Sweden**			
**M = Male**	**Respond %**		**General pop%**		**Respond %**		**General pop%**		**Respond %**		**General pop%**		**Respond %**		**General pop%**		**Respond %**		**General pop%**	
**F = Female**	**M**	**F**	**M**	**F**	**M**	**F**	**M**	**F**	**M**	**F**	**M**	**F**	**M**	**F**	**M**	**F**	**M**	**F**	**M**	**F**
**Marital status** ^**a**^																				
Married/cohab	87.5		77.6		88.5		79.7		87.9		72.2		87.3		81.0		88.1		76.6	
Single	1.6	10.9	3.9	18.5	1.4	10.1	2.7	17.6	0.7	11.3	2.5	25.3	2.2	10.5	3.2	15.8	1.6	10.3	4.8	18.6
**Educational level** ^**b**^																				
Lower levels	45.8	56.0	71.8	64.4	59.2	60.1	79.1	74.6	47.7	43.6	73.3	63.8	43.6	38.2	69.9	65.5	53.9	46.1	66.5	58.9
University level	54.2	44.0	28.2	35.6	40.8	39.9	20.9	25.3	52.3	56.4	26.7	36.2	56.4	61.8	30.1	34.5	52.1	53.9	33.5	41.1
**Country of birth** ^**c**^																				
Nordic	89.0	94.4	90.3	90.6	97.4	98.8	95.0	95.3	92.2	96.9	91.2	91.0	88.6	90.5	89.8	90.4	86.4	89.5	87.7	87.7
Non-Nordic	11.0	5.6	9.7	9.4	2.6	1.2	4.3	4.1	7.8	3.1	8.8	9.0	11.4	9.5	10.2	9.6	13.6	10.5	12.3	12.3

^a^general population: parents with children aged 0-17 living at home.

^b^general population: DK people aged 25-69, FI people aged 25-69, IS people aged 25-74, NO people >25, SE people aged 25-74.

^c^general population: the whole population.

*Source: National statistical institutes.*

Finally, the questionnaire was addressed to the parent regarded as the primary caregiver, of whom the large majority were mothers, reflecting the social norm of mothers taking the main responsibility in childcare. Accordingly, the fathers participating in the study should be considered norm breaking and the results of the study only valid for parents who are the child’s primary caregiver, and not mothers and/or fathers in general. The Nordic variation in the prevalence of experienced time pressure is an interesting issue, at present without any firm explanation. As we have discussed in another article [18], Danes actually do experience stress in everyday life according to previous studies [60,61], just as psychologists describe high pace and multiple roles as main challenges in everyday life of Danish parents [62]. Similarly to our results, however, a lower proportion of Danish workers reported work-related stress as compared to the Nordic and European average [63]. Hence, further research on the prevalence, origin and impacts of parents’ time pressure is needed.

## Conclusions

This study found that parents’ subjective time pressure was associated with increased mental health problems among children and adolescents. Given that time pressure is a growing feature of modern societies, such as those in the Nordic countries, the results might contribute to an explanation as to why mental health problems are common among children in the Nordic countries in spite of otherwise favourable conditions for child health and development. Additional research on the linkage between parents’ experienced time pressure and children’s and adolescents’ mental health problems is needed to confirm the novel findings of this study. Further research is also needed regarding the mechanisms between parents’ time pressure and children’s mental health problems as well as concerning the gendered patterns of mental health problems among children and adolescents.
